# A Deng Entropy-Based Heuristic Method to Determine Discounting Coefficient in Dempster-Shafer Evidence Fusion

**DOI:** 10.3390/e28070806

**Published:** 2026-07-15

**Authors:** Siyao Huang, Yong Deng

**Affiliations:** 1 College of Computer Science and Artificial Intelligence, Southwest Minzu University, Chengdu 610225, China; 202330903105@stu.swun.edu.cn; 2 Institute of Fundamental and Frontier Science, University of Electronic Science and Technology of China, Chengdu 611731, China

**Keywords:** Dempster-Shafer evidence theory, conflict management, Deng entropy, sigmoid function

## Abstract

Conflict management is crucial in information fusion. One of the efficient algorithms to address conflicting data fusion is discounting method. However, how to determine the discounting coefficient in conflict management remains an open issue. A heuristic method to determine discounting coefficient is presented based on Deng entropy and sigmoid function. Where Deng entropy quantifies the uncertainty of evidence and the sigmoid function maps it to a reasonable coefficient range. The effectiveness of the proposed method is illustrated by numerical example and real application. Compared with existing methods to determine discounting coefficients, the proposed method shows promising performance in the analyzed examples and is simple to implement.

## 1. Introduction

Conflicts in evidence are very common, especially when handling uncertain and insufficient data. Thus, conflict management is inevitable and extremely important in numerous fields. In practical scenarios, data obtained from different sensors is typically uncertain or has high conflict degree, which can significantly affect the performance of information fusion. How to deal with conflicts effectively has become a major challenge in information fusion.

To address this issue, Dempster-Shafer (D-S) evidence theory has been broadly adopted [1,2,3]. Evidence theory offers a mathematical foundation for fusing multiple bodies of evidence [4,5,6]. It is widely adopted in uncertainty analysis [7,8,9], multi-sensor data fusion [10,11], decision making [12,13], multi-sensor fault diagnosis [14,15,16] and underwater communication [17,18,19,20]. In addition, recent studies have further expanded the applications, such as water resource allocation [21], pedestrian dynamics [22], and transport infrastructure analysis [23]. Therefore, D-S evidence theory is effective, especially in uncertain conditions. A novel entropy function enables the processing of conflict sensor inputs without implementing any prior information [24]. While evidential correlation coefficient (ECC) in decision-making was proposed to apply in the field of fault diagnosis [25]. Evidence theory was also widely used in landslide susceptibility [26], land cover [27] and identification of influential nodes [28].

Notably, direct analysis of the basic probability assignment (BPA) often fails to yield profound insights in most conditions. As dominant high probability components tend to obscure the subtle patterns and latent features embedded within the data. In this way, evidence theory may produce counterintuitive and unconvincing results when the collected evidence presents high conflicts, significantly limiting its applicability in practical engineering problems [29].

Realizing this challenge, researchers have presented various methods to better manage the conflicts. Smets [30,31] introduced a foundational framework for quantifying the informational value of evidence. Murphy [32] and Yager [33] also proposed alternative combination strategies. Several of the earliest systematic treatments of how conflict belief functions interact during the evidence combining process were offered. Recently, the discrepancy between belief functions and support fusion weight allocation can be quantified through the implementation of divergence-based measures. For instance, enhanced belief divergence [34], reinforced final belief divergence [35], belief exponential divergence [36], and complex belief divergence [37] offer distinct perspectives on measuring evidential dissimilarity. Furthermore, generalized f-divergence [38] extends the applicability of divergence measures to belief functions. While fractal belief Renyi divergence [39] and intuitionistic fuzzy sets [40] provide additional tools for addressing conflict in pattern classification. Apart from divergence, approaches focusing on similarity received extensive concern as well. Belief logarithmic similarity [41], evidential similarity based on tanimoto measure [42] and a novel sine similarity [43] have been developed to evaluate the conflict degree between evidence. In addition, an enhanced pignistic probability function-based combination approach has been proposed to mitigate conflicting evidence and enhance fusion performance [44].

Among numerous methods, fusion approaches based on discounting coefficients still work effectively. Consequently, various improved approaches introduced discounting coefficients based on D-S theory reduce the influence of unreliable evidence. Common discounting coefficients such as distance and entropy are widely adopted to alleviate the conflict between different sets of evidence [45,46,47]. For example, distance-based measure for D numbers has been developed and subsequently utilized in product engineering applications [48]. Based on distance [49], modified average method was proposed to combine belief function A modified average method based on distance [49] was proposed to combine belief function [50]. Similarly, a method of rectifying the distance based on BJS divergence and penalty coefficient is proposed [51]. Besides, to quantify uncertainty in Dempster-Shafer evidence theory, a generalized extension of Shannon entropy is proposed [52]. While Renyi-Deng entropy is used by generalized belief entropy to detect contradicting evidence [53]. In addition, belief Tanimoto coefficient measures the consistency between bodies of evidence by considering both their length and direction [54]. Distance-based methods evaluate the reliability of evidence by measuring the distance between basic probability assignments. Whereas entropy-based approaches quantify evidence uncertainty using information measures. Besides, Information fusion fault diagnosis method [55] and new dissimilarity measure [56] were proposed based on discounting factors.

However, most of these methods either rely on a single perspective or fail to consider the influence between uncertainty and conflict, which may limit their effectiveness in highly conflicting conditions. How to ensure the discounting coefficient rationally remains an unresolved issue, constituting the focus of this study. This paper proposes a heuristic Deng entropy-based approach aiming at determining the discounting coefficient to address this issue. Existing discounting coefficients may increase the computational complexity or introduce extra information. With the application of Deng entropy and sigmoid function, the belief distribution is automatically redistributed. It performs a discounting effect on the evidence itself. Therefore, the discounting coefficient is data-driven, originated from the evidence itself without additional information. Finally, by some numerical examples and real applications, the proposed method achieves satisfactory performance in terms of fusion accuracy, resistance to noisy evidence, and convergence performance.

The rest of this paper is structured as follows. Dempster-Shafer evidence theory and some relevant techniques are introduced in Section 2. The heuristic discounting method is proposed and discussed in Section 3. In Section 4, a numerical example, real application in target recognition and real dataset are conducted to illustrate the discounting method. Conclusions are analyzed in Section 5.

## 2. Preliminaries

Several basic concepts of Dempster-Shafer evidence theory, discounting coefficient, Deng entropy and sigmoid function are briefly introduced. Discounting coefficient is incorporated to quantify the credibility of evidence sources. Furthermore, Deng entropy is presented as an effective measure for quantifying the uncertainty of mass functions, and the sigmoid function is discussed for its role in modeling nonlinear transformations.

### 2.1. Dempster-Shafer Evidence Theory

The Dempster-Shafer evidence theory provides a mathematical structure for reasoning under ambiguity and imprecision. Unlike traditional probabilistic models, it can effectively manage incomplete and conflict information even without depending on prior probabilities [57]. Owing to these advantages, it provides a strong framework for managing and modeling uncertainty in complex systems.

Evidence theory defines a frame of discernment as [3]:

(1) $$\Theta =\{\theta_{1},\theta_{2},\ldots ,\theta_{n}\}$$

where $\theta_{i}$ denotes a hypothesis that is exhaustive of all the evidence and mutually exclusive. A mapping is known as a BPA [3]:

(2) $$m:2^{\Theta }\to \left[0,1\right],$$

which satisfies [3]:

(3) $$m\left(\emptyset \right)=0,\;\sum_{A\subseteq \Theta }m\left(A\right)=1.$$

The belief which precisely committed to the proposition *A* is quantified by m(A). Focal elements are subsets *A* with m(A)>0. When two evidence sets $m_{1}$ and $m_{2}$ are available, Dempster proposed the combination rule as follows [3]:

(4) $$m\left(\emptyset \right)=0,\;m\left(A\right)=\frac{\sum_{B\cap C=A}m_{1}\left(B\right)\;m_{2}\left(C\right)}{1-K}$$

where the conflict coefficient *K* is defined as [3]:

(5) $$K=\sum_{B\cap C=\emptyset }m_{1}\left(B\right)\;m_{2}\left(C\right)$$

here, *K* evaluates the conflict degree between evidence. A higher *K* indicates stronger inconsistency between evidence, leading to counterintuitive or unreliable fusion results in high-conflict situations probably.

### 2.2. Discounting Coefficients

When a piece of evidence is not fully reliable, its degree of support should be weakened by a discounting process. The discounting coefficient α∈[0,1] is used to adjust the BPA of a source according to its reliability in the D-S evidence theory [6]. Defined on space Θ, assume a belief function Bel(·) with α∈[0,1] denoting the discount rate. The discounted belief function is formulated as [6]:

(6) $$Bel^{\alpha }\left(A\right)=\left(1-\alpha \right)Bel\left(A\right),\;\forall A\subset \Theta .$$

In practical applications, the discounted mass function denoted by $m^{\alpha }$ [6]:

(7) $$m^{\alpha }\left(A\right)=\left(1-\alpha \right)\;m\left(A\right),\;\forall A\subset \Theta ,A\neq \Theta$$

(8) $$m^{\alpha }\left(\Theta \right)=\left(1-\alpha \right)\;m\left(\Theta \right)+\alpha$$

where α represents the uncertainty or unreliability level in evidence, *m* denotes the original mass function. This mechanism redistributes the discounted mass to Θ, thereby weakening the influence of unreliable evidence before applying Dempster’s combination rule.

### 2.3. Deng Entropy

A measure of BPA uncertainty known as Deng entropy [52] was proposed inspired by the Shannon entropy [58]. Deng entropy extends the classical Shannon entropy concept in frame of belief functions. Assuming a frame of discernment Θ and a mass function denoted as *m*, Deng entropy is expressed as [52]:

(9) $$E_{d}\left(m\right)=-\sum_{A\subseteq \Theta ,A\neq \emptyset }m\left(A\right)\log_{2}\frac{m(A)}{2^{\left|A\right|}-1}$$

where the focal element is *A*, with |A| denoting its cardinality. It takes into account not only the mass value m(A) but also the size of the focal element. By taking these two factors into account, Deng entropy can explain the randomness and non-specificity observed in the evidence. Larger entropy represents a higher uncertainty, while smaller entropy represents a more specific and informative evidence.

### 2.4. Sigmoid Function

The sigmoid function is widely utilized in statistics and neural networks. Which is defined as [59]:

(10) $$\sigma \left(x\right)=\frac{1}{1+e^{-x}}$$

where x∈(−∞,+∞) is reflected into the interval (0,1). Due to its continuity, differentiability and strict monotonicity, the sigmoid function offers a smooth transition from its lower to upper bounds. Owing to its nonlinearity, it works particularly well for mapping unbounded values to bounded coefficients and retaining input ratio information. This feature keeps the patterns of variation of the degree of evidence conflict intact while allowing the sigmoid function to fit to the range of evidence weighting coefficients, preventing the coefficients from taking on nonsensical extreme values.

## 3. Proposed Method

In order to measure uncertainty and determine dependability, several earlier works have suggested entropy-based weighting methods in evidence theory as shown in Table 1. Nevertheless, these techniques frequently depend on heuristic normalizations or indirect transformations.

In uncertainty measurement, Deng entropy [52] operates as an efficient instrument for the quantification of uncertainty of BPAs, which enables a more reliable decision-making applications in various fields. To determine the discounting coefficient, a heuristic approach using Deng entropy [52] and sigmoid function [59] is presented in this section. The overall procedure consists of four key phases: discounting based on Deng entropy and sigmoid function, discounting of BPAs, properties of the proposed method and combination based on discounting coefficient.

### 3.1. Discounting Based on Deng Entropy and Sigmoid Function

To further transform the uncertainty into reliable weight for evidence, the entropy value is projected to a discounting coefficient through a sigmoid function [59]. Using the result of Deng entropy [52] as *x* in the sigmoid function. Specifically, the mapping procedure takes the value of Deng entropy as input. Through the transformation of the sigmoid function, it enables abstract entropy metrics into discounting factors. Therefore, it realizes quantitative and reliable evaluation, along with reasonable adjustment.

**Definition** **1.** *Let $m_{i}$ denotes a BPA on space* Θ*. The discounting in each evidence is defined as follows:*

(11) $$w_{i}=\frac{1}{1+e^{-E_{d}\left(m_{i}\right)}}$$

*where $E_{d}\left(m_{i}\right)$ represents the uncertainty of $m_{i}$, evaluated by applying Deng entropy [52]. The volume of information is quantified systematically. It should be pointed out that the purpose of using entropy is to measure uncertainty, rather than quantify the degree of conflict between evidence. Although Deng entropy does not measure conflict directly, the value of Deng entropy can heuristically serve as an indicator of the cause of conflict to some degree, reflecting the possibility of causing conflicts. Meanwhile, entropy reflects the information volume and the flexibility of evidence to some extent. Evidence with larger entropy generally carries greater information volume and flexibility. Therefore, higher weights should be assigned to such evidence. Conversely, overconfident evidence should be given lower weight, which mitigates the adverse effects of highly conflicting evidence during the fusion process reasonably. Based on this, the discounting coefficient can be constructed to adjust each piece of evidence accordingly before the fusion process. In this way, it avoids treating all evidence equally and establishing a reasonable basis for subsequent adaptive evidence fusion.*

### 3.2. Discounting of BPAs

**Definition** **2.** *Applying the discounting coefficient to each evidence, the BPAs are redistributed as follows:*

(12) $$m_{i}^{\prime }\left(A\right)=w_{i}m_{i}\left(A\right),\;\forall A\subset \Theta$$

(13) $$m_{i}^{\prime }\left(\Theta \right)=w_{i}m_{i}\left(\Theta \right)+\left(1-w_{i}\right)$$

Using the sigmoid function, the entropy value is smoothly mapped into (0,1), serving as the discounting coefficient together. Based on this discounting coefficient, the BPAs are redistributed, leading to a more reasonable belief assignment. Moreover, the adverse influence of highly conflicting evidence is effectively alleviated by reducing its contribution to the fusion process. As a result, the overall combination becomes more stable, while evidence with larger information volume and flexibility is allowed to have a more dominant impact in the final decision.

### 3.3. Properties of the Proposed Method

To analyze the mathematical properties of this operation more rigorously, the following properties are obtained.

**Normalization:** After discounting, each focal element A⊂Θ,A≠Θ is transformed as $m_{i}^{\prime }\left(A\right)=w_{i}\cdot m_{i}\left(A\right)$, while the mass assigned to Θ is updated as $m_{i}^{\prime }\left(\Theta \right)=w_{i}m_{i}\left(\Theta \right)+\left(1-w_{i}\right)$. Therefore,

(14) $$\sum_{A\subseteq \Theta }m_{i}^{\prime }\left(A\right)=\sum_{A\subset \Theta ,A\neq \Theta }w_{i}m_{i}\left(A\right)+w_{i}m_{i}\left(\Theta \right)+\left(1-w_{i}\right)=1,$$

which guarantees that the BPA remains normalized.

**Monotonicity:** The sigmoid mapping ensures that $w_{i}\in \left(0,1\right]$, which follows that $m_{i}^{\prime }\left(A\right)=w_{i}m_{i}\left(A\right)\leq m_{i}\left(A\right)$ for all A≠Θ, while $m_{i}^{\prime }\left(\Theta \right)=w_{i}m_{i}\left(\Theta \right)+\left(1-w_{i}\right)\geq m_{i}\left(\Theta \right)$. This indicates that the proposed method reduces the belief assigned to specific hypotheses and reallocates it to Θ.

**Boundedness:** Given $w_{i}\in \left(0,1\right]$ and $m_{i}\left(A\right)\in \left[0,1\right]$, $m_{i}^{\prime }\left(A\right)$ is guaranteed to satisfy $m_{i}^{\prime }\left(A\right)\in \left[0,1\right]$ for all A⊆Θ, thus preserving boundedness.

**Time complexity:** Let *k* denote the number of BPAs and *n* be the element count in Θ. In the worst case, each BPA contains up to $2^{n}$ focal elements. The proposed method involves Deng entropy computation, discounting and Dempster’s combination. Both entropy and discounting require scanning all focal elements with a complexity of $O(k\cdot 2^{n})$. The dominant cost arises from the evidence combination stage, where Dempster’s rule requires pairwise operations over focal elements, leading to a complexity of $O(4^{n})$ for combining two BPAs and $O(k\cdot 4^{n})$ for sequential fusion of *k* BPAs. Therefore, the overall computational complexity is $O(k\cdot 4^{n}+k\cdot 2^{n})$, which can be simplified as $O(k\cdot 4^{n})$. Compared with the classical Dempster-Shafer combination method [6], the proposed method does not increase the computational complexity, but only introduces an additional linear cost.

### 3.4. Combination Based on Discounting Coefficient

After Discounting BPAs, the influence of highly conflicting evidence is weakened. A more stable representation of the collective information can be acquired. Applying Dempster’s combination rule [6] to the two discounted BPAs $m_{1}^{\prime }$ and $m_{2}^{\prime }$, the fusion process is obtained as follows:

(15) $$m\left(A\right)=\frac{\sum_{B\cap C=A}m_{1}^{\prime }\left(B\right)m_{2}^{\prime }\left(C\right)}{1-K},\;A\neq \emptyset$$

The fusion process operates n−1 times to obtain the results. Through the discounting method along with classical D-S fusion procedure, the proposed approach provides an effective way to handle conflicting evidence. In particular, the adjustment of the evidence is entirely data-driven. Based on the original information structure of the BPA, the evidence weights are adaptively adjusted to reduce the influence of highly conflicting evidence.

The general framework of the discounting method is summarized in Algorithm 1. It heuristically combined the application of Deng entropy and sigmoid function, which serves to discount the original evidence and reduce interference from highly conflicting information.
**Algorithm 1:** Proposed Method
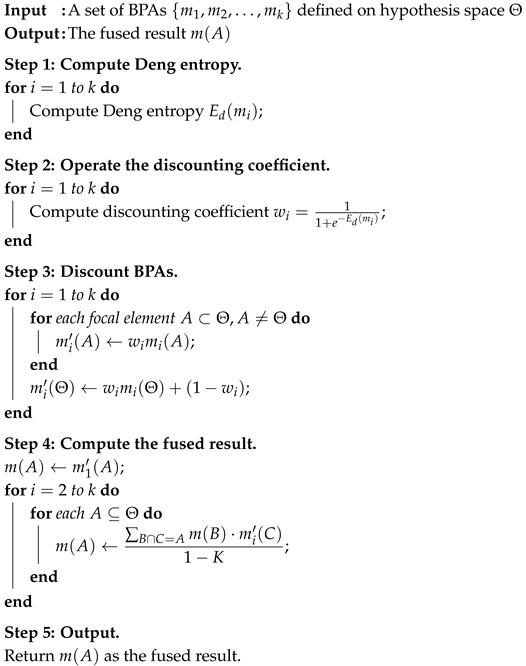


## 4. Numerical Examples

In this section, detailed computational procedure of the discounting method in numerical example, application and real dataset are illustrated.

### 4.1. Computational Process of the Proposed Method

To demonstrate the application in the discounting measure, a hypothetical example is provided. Assume *A* is the true target for a multi-sensor automatic target identification system. Five sets of evidence were collected by the system in five diverse sensors, which is shown below in Table 2.

The computational process is detailed in four steps.

**Step 1:** Computation of Deng entropy.

For a given set of evidences $\left\{\left(R_{1},m_{1}\right),\left(R_{2},m_{2}\right),\ldots ,\left(R_{k},m_{k}\right)\right\}$, in each evidence, the uncertainty of $m_{i}$ is quantified by Deng entropy, which is computed as:

$$\begin{array}{cc} E_{d}\left(m_{1}\right) & =-(0.49\log\frac{0.49}{2-1}+0.21\log\frac{0.21}{2-1}+0.3\log\frac{0.3}{2-1})=1.4982,\\ E_{d}\left(m_{2}\right) & =0.2864,\\ E_{d}\left(m_{3}\right) & =1.9168,\\ E_{d}\left(m_{4}\right) & =1.8532,\\ E_{d}\left(m_{5}\right) & =1.9527. \end{array}$$

**Step 2:** Calculation of the discounting coefficient.

Discounting of each evidence is computed below:

$$\begin{array}{cc} w_{1} & =\frac{1}{1+e^{-1.4982}}=0.8173,\\ w_{2} & =0.5711,\\ w_{3} & =0.8718,\\ w_{4} & =0.8645,\\ w_{5} & =0.8757. \end{array}$$

**Step 3:** Discount the BPAs.

With the discounting coefficient, the evidence is reallocated as follows:

$$\begin{array}{cc} m_{1}\left(A\right) & =0.8173\times 0.49=0.4005,m_{1}\left(B\right)=0.8172\times 0.21=0.1716,\\ m_{1}\left(C\right) & =0.8173\times 0.3=0.2452,m_{1}\left(ABC\right)=1-0.8154=0.1827,\\ m_{2}\left(B\right) & =0.5426,m_{2}\left(C\right)=0.0286,m_{2}\left(ABC\right)=0.4288,\\ m_{3}\left(A\right) & =0.4621,m_{3}\left(B\right)=0.0610,m_{3}\left(AC\right)=0.3487,m_{3}\left(ABC\right)=0.1282,\\ m_{4}\left(A\right) & =0.4755,m_{4}\left(B\right)=0.0432,m_{4}\left(AC\right)=0.3458,m_{4}\left(ABC\right)=0.1355,\\ m_{5}\left(A\right) & =0.5079,m_{5}\left(B\right)=0.0963,m_{5}\left(AC\right)=0.2540,m_{5}\left(ABC\right)=0.1418. \end{array}$$

Applying the Dempster’s rule [6], the obtained evidence is further combined. The fusion result obtained in Table 3. Additionally, according to one reviewer’s suggestion, the fusion results obtained using the normalized Deng entropy are presented in Table 4 for comparison.

**Figure 1:**
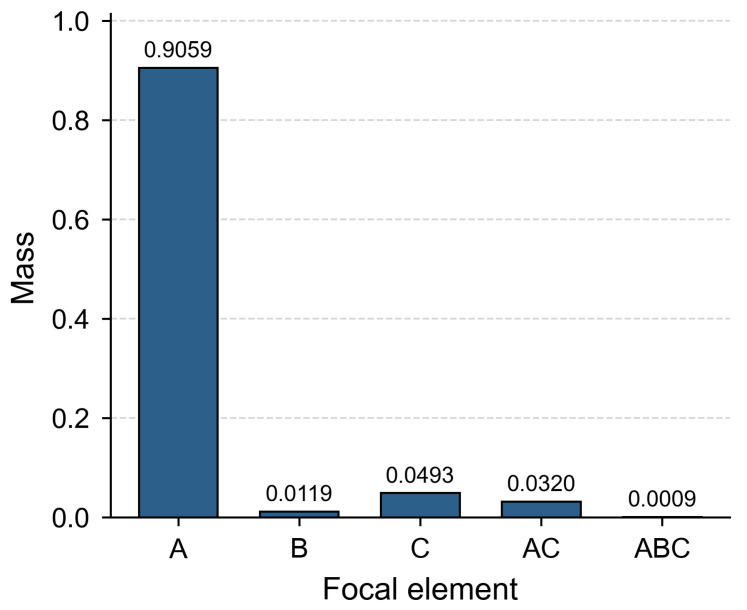
Fusion results obtained by the discounting method.

As clearly demonstrated in Table 3 and Figure 1, the fusion recognizes the target successfully, exhibiting a clear and consistent trend as more evidence sources are integrated. After redistributing the BPAs, evidence with high conflicts is gradually suppressed. By discounting based on both the sigmoid function and uncertainty, the belief assigned to elements becomes more balanced. In the following fusion steps, the belief mass allocated to hypothesis *A* increases steadily. In Particular, the belief assigned to the singleton focal element *B* is effectively suppressed as additional evidence is fused, indicating reduced uncertainty. These obtained results indicate that the discounting method can progressively improve the decision confidence, keeping consistent fusion performance with multiple sources of evidence.

### 4.2. Real Application in Radar Observations

To assess the efficiency of the discounting approach in practical settings, we carried out applications involving 30 practical BPAs collected from a radar sensor. Each BPA represents an individual sensing output, where the mass values reflect the support assigned to different hypotheses. Among these observations, six of the evidence sets are conflicting, appearing in the 6th, 12th, 18th, 23rd, 28th and 30th evidence sets. The main purpose is to evaluate computing efficiency and stability of the fusion outcomes, especially the effectiveness under abnormal or conflicting inputs. It provides a crucial theoretical support and experimental basis for the promotion and application of this method in practical engineering scenarios such as intelligent perception and target decision-making.

**Figure 2:**
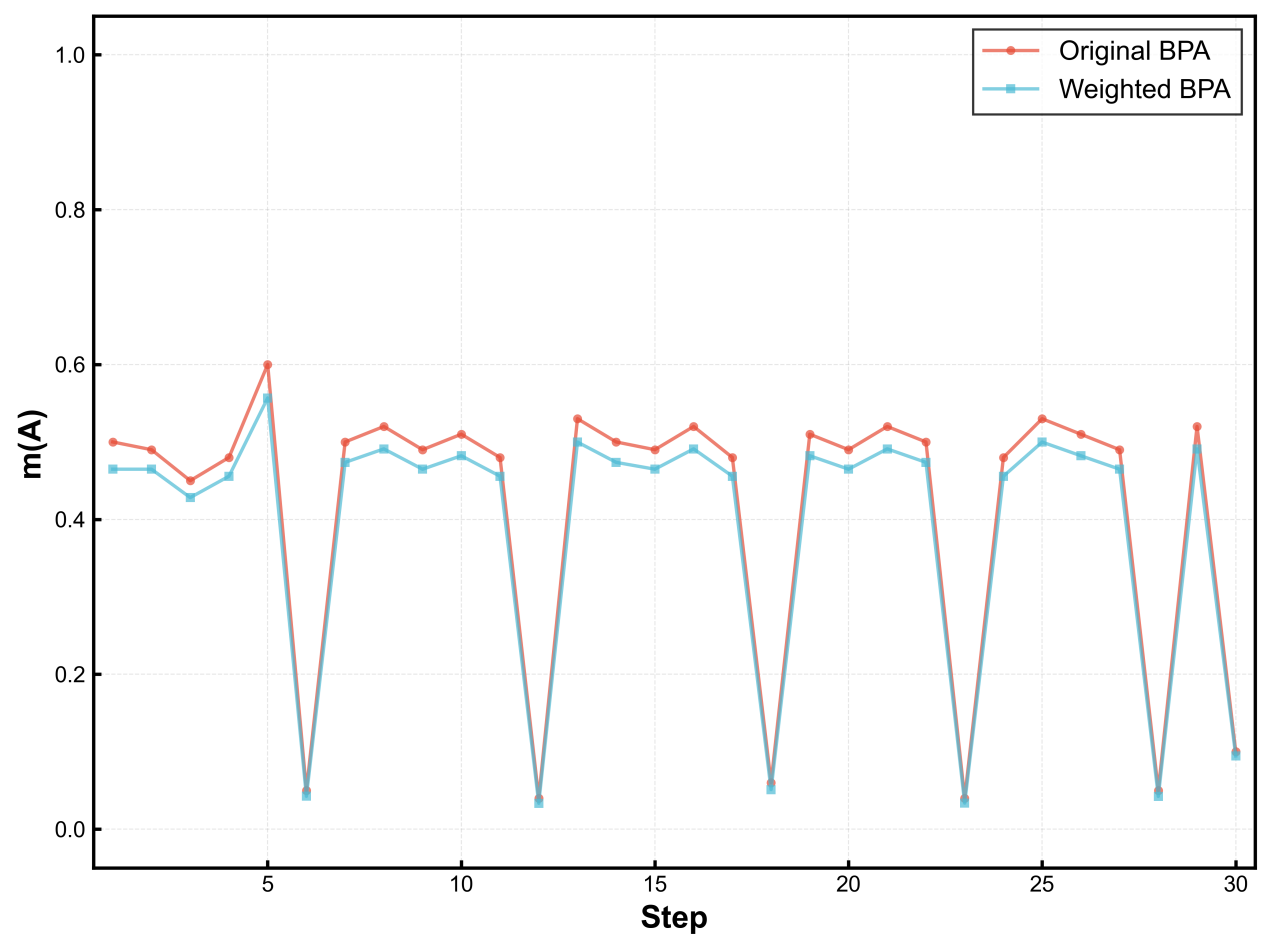
Comparison of the BPA in target set.

**Figure 3:**
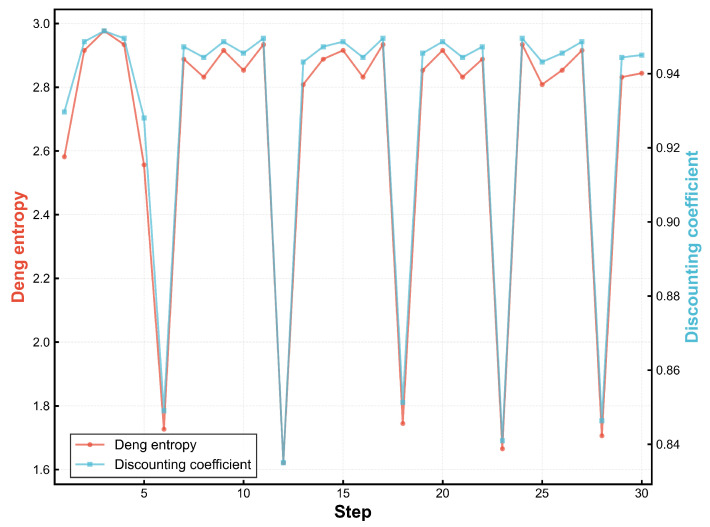
Value of discounting coefficient and entropy.

**Figure 4:**
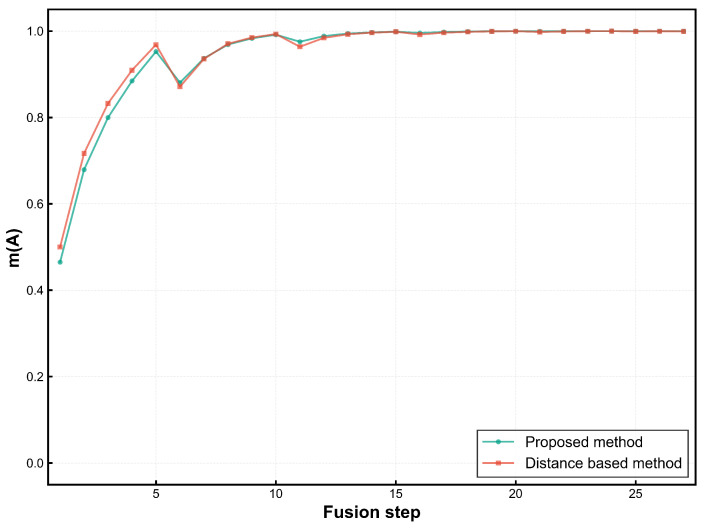
Comparison of fusion result.

Given BPA values of 30 evidence sets under discernment Θ={A,B,C} in Table 5, while the target set is *A*. Figure 2 illustrates the BPA of *A* in different process. Figure 3 reveals values of Deng entropy and sigmoid-based discounting coefficient. Figure 4 presents the fusion results and evolution process of the target set *A*.

According to Figure 2, the trend of original BPA and discounting BPA is overall consistent. It indicates that the proposed discounting method ensures the stability in the evidence processing. From Figure 3, it is clear that the uncertain degree of evidence is recognized effectively. Meanwhile, the discounting coefficient of conflicting evidence decreases. This is because higher entropy indicates richer but more diversified information, which is given greater weight. Conversely, lower entropy results in a smaller weight due to its relatively limited flexibility during the fusion process. It mitigates the effect on the fusion process, avoiding the error caused by the dominance of conflicting evidence in classical fusion methods effectively. Finally in Figure 4, compared with the distance based method [50], the fusion result shows a similar trend across both methods that *A* increases rapidly in the following steps and reaches its maximum. It demonstrates their ability to correctly identify the target. In addition, with low computational complexity, the proposed method has the capacity to progressively reinforce the correct target and achieve stable identification, especially under uncertain and highly conflicting condition.

**Figure 5:**
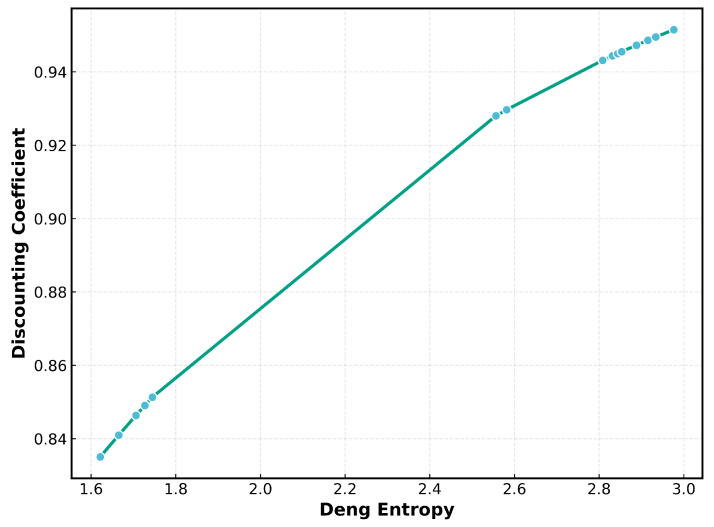
Variation of discounting coefficient with entropy.

**Figure 6:**
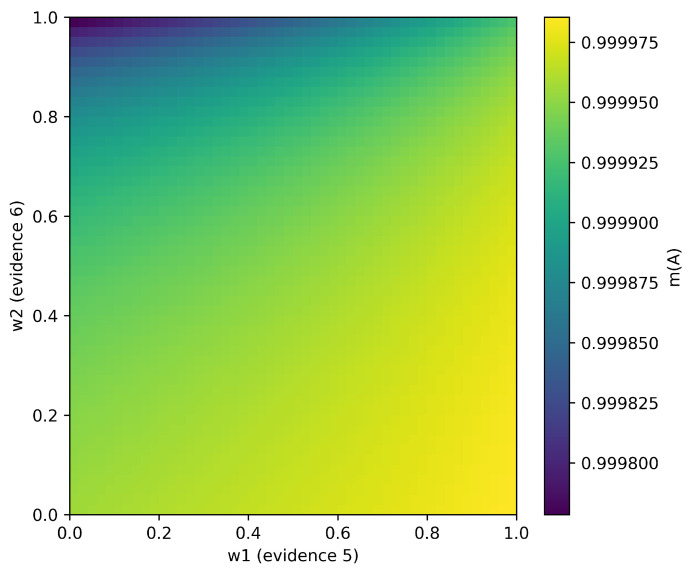
Fusion sensitivity heatmap.

To further evaluate the characteristic of the proposed method, detailed analyses are conducted. As demonstrated in Figure 5, variations in entropy values have a direct impact on the discounting coefficients. The discounting coefficient increases monotonically as entropy increases, indicating higher uncertainty in the evidence results in a larger discounting factor. In Figure 6, belief of m(A) remains stable, which indicates that the proposed method can effectively alleviate the influence of conflicting evidence. It can effectively moderate the influence of evidence and stabilize fusion performance.

### 4.3. Application in Iris Dataset

To further validate the generalization of the proposed method, the Iris dataset [63] is applied. Within the discernment Θ, three hypotheses are defined as A,B,C corresponding to the Iris species Setosa, Versicolor and Virginica respectively.

Using 50 samples with the target *A*, BPAs are constructed by computing the Euclidean distance between each sample. The resulting similarities are then normalized to obtain the initial belief distributions over the three hypotheses. To model realistic measurement uncertainty, each BPA component is perturbed by ±50%. The perturbation is intended as an illustrative stress test rather than a comprehensive validation of robustness.

**Figure 7:**
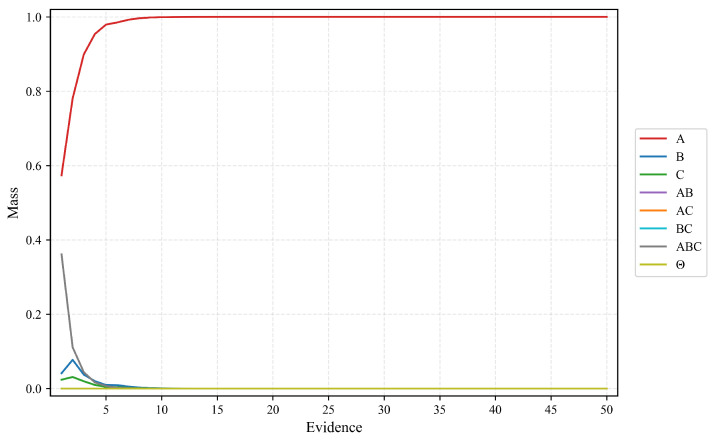
Fusion result on Iris dataset.

As illustrated in Figure 7, even with additional disturbance introduced into the evidence, the proposed method still achieves stable and effective identification of the target, demonstrating reliable decision-making performance under conflicts.

Although the proposed method demonstrates satisfactory performance in both numerical examples and real applications, several limitations should be acknowledged. When all evidence sources are highly unreliable, the proposed discounting strategy may unable to compensate for the absence of reliable information, and the fusion performance may deteriorate accordingly. Additionally, highly conflicting evidence may occasionally contain valuable information. These issues will be investigated in future work through more adaptive discounting strategies and computational optimization.

## 5. Conclusions

In this paper, a Deng entropy-based heuristic approach is presented to determine discounting coefficient in evidence fusion, which can be applied to conflict management. The proposed method exhibits many specific properties compared to existing discounting strategies. Uniquely, the discounting process is conducted through Deng entropy and sigmoid function, where the evidence is modified based on its weight in uncertainty without any other information. Therefore, the entire procedure is data-driven, which is determined only by the given sets of evidence, making the method more adaptive. Meanwhile, the proposed method is further implemented in real application. The results show that the proposed method exhibits efficiency even the collected evidence highly conflicting with each other. The approach is simple to calculate. These properties provide a desirable perspective for evidence discounting for managing conflicts in data fusion. Nevertheless, broader validation using repeated experiments, statistical tests, confidence intervals and systematic comparisons remains necessary.

## Figures and Tables

**Table 1:** Prior weighting methods based on entropy.

Methods	Expression	Maximum Distribution	Maximum
Li & Xiao [60]	$\begin{array}{cc} W_{i} & =\frac{E_{d}\left(m_{i}\right)}{\left|E_{d}\left({\bar{m}}_{i}\right)-E_{d}\left(m_{i}\right)\right|} \end{array}$	m(Θ)=1	+∞
Li et al. [61]	$\begin{array}{cc} \omega_{i} & =\frac{{\mathrm{Cred}}_{i}}{\sum_{l=1}^{n}{\mathrm{Cred}}_{l}} \end{array}$	$m\left(\theta_{i}\right)=\frac{1}{\left|\Theta \right|}$	$\omega_{i}=\frac{1}{n}$
Wang et al. [62]	$\begin{array}{cc} DEn_{i} & =\frac{DE(m_{i})}{\sum_{l=1}^{n}DE\left(m_{l}\right)}\\ Crdmm_{i} & =\frac{Crd_{i}\cdot DEn_{i}}{\sum_{l=1}^{n}\left(Crd_{l}\cdot DEn_{l}\right)} \end{array}$	$m\left(\theta_{i}\right)=\frac{1}{\left|\Theta \right|}$	$Crdmm_{i}=\frac{1}{n}$

**Table 2:** Evidence sets from five sensors.

Evidence	m({A})	m({B})	m({C})	m({A,C})
$R_{1}$	0.49	0.21	0.30	0
$R_{2}$	0	0.95	0.05	0
$R_{3}$	0.53	0.07	0	0.40
$R_{4}$	0.55	0.05	0	0.40
$R_{5}$	0.58	0.11	0	0.29

**Table 3:** Results of the evidence combination.

	$m_{1},m_{2}$	$m_{1},m_{2},m_{3}$	$m_{1},m_{2},m_{3},m_{4}$	$m_{1},m_{2},m_{3},m_{4},m_{5}$
The proposed method	m(A) = 0.2712	m(A) = 0.5709	m(A) = 0.7892	m(A) = 0.9059
	m(B) = 0.4198	m(B) = 0.1593	m(B) = 0.0392	m(B) = 0.0119
	m(C) = 0.1853	m(C) = 0.1618	m(C) = 0.1029	m(C) = 0.0493
	m(A,C) = 0	m(A,C) = 0.0790	m(A,C) = 0.0635	m(A,C) = 0.0320
	m(A,B,C) = 0.1237	m(A,B,C) = 0.0290	m(A,B,C) = 0.0052	m(A,B,C) = 0.0009

**Table 4:** Results of the evidence combination after normalization.

	$m_{1},m_{2}$	$m_{1},m_{2},m_{3}$	$m_{1},m_{2},m_{3},m_{4}$	$m_{1},m_{2},m_{3},m_{4},m_{5}$
The proposed method after normalization	m(A) = 0.1826	m(A) = 0.3693	m(A) = 0.5577	m(A) = 0.7076
	m(B) = 0.4238	m(B) = 0.2762	m(B) = 0.1547	m(B) = 0.0927
	m(C) = 0.1318	m(C) = 0.1187	m(C) = 0.0963	m(C) = 0.0669
	m(A,C) = 0	m(A,C) = 0.0910	m(A,C) = 0.1188	m(A,C) = 0.0980
	m(A,B,C) = 0.2618	m(A,B,C) = 0.1448	m(A,B,C) = 0.0725	m(A,B,C) = 0.0348

**Table 5:** 30 sets data from a radar sensor.

BPA	{A}	{B}	{C}	{A,B}	{A,C}	{B,C}	{A,B,C}	BPA	{A}	{B}	{C}	{A,B}	{A,C}	{B,C}	{A,B,C}
$m_{1}$	0.50	0.23	0.02	0.06	0.06	0.02	0.11	$m_{16}$	0.52	0.11	0.04	0.11	0.08	0.04	0.10
$m_{2}$	0.49	0.13	0.05	0.11	0.07	0.05	0.10	$m_{17}$	0.48	0.14	0.05	0.11	0.07	0.05	0.10
$m_{3}$	0.45	0.17	0.04	0.12	0.08	0.04	0.10	$m_{18}$	0.06	0.75	0.04	0.04	0.03	0.03	0.05
$m_{4}$	0.48	0.14	0.05	0.11	0.07	0.05	0.10	$m_{19}$	0.51	0.12	0.04	0.11	0.08	0.04	0.10
$m_{5}$	0.60	0.07	0.04	0.07	0.08	0.04	0.10	$m_{20}$	0.49	0.13	0.05	0.11	0.07	0.05	0.10
$m_{6}$	0.05	0.76	0.03	0.04	0.02	0.03	0.07	$m_{21}$	0.52	0.11	0.04	0.11	0.08	0.04	0.10
$m_{7}$	0.50	0.12	0.05	0.11	0.08	0.04	0.10	$m_{22}$	0.50	0.12	0.05	0.11	0.08	0.04	0.10
$m_{8}$	0.52	0.11	0.04	0.11	0.08	0.04	0.10	$m_{23}$	0.04	0.77	0.04	0.04	0.02	0.02	0.07
$m_{9}$	0.49	0.13	0.05	0.11	0.07	0.05	0.10	$m_{24}$	0.48	0.14	0.05	0.11	0.07	0.05	0.10
$m_{10}$	0.51	0.12	0.04	0.11	0.08	0.04	0.10	$m_{25}$	0.53	0.10	0.04	0.11	0.08	0.04	0.10
$m_{11}$	0.48	0.14	0.05	0.11	0.07	0.05	0.10	$m_{26}$	0.51	0.12	0.04	0.11	0.08	0.04	0.10
$m_{12}$	0.04	0.78	0.03	0.03	0.02	0.02	0.08	$m_{27}$	0.49	0.13	0.05	0.11	0.07	0.05	0.10
$m_{13}$	0.53	0.10	0.04	0.11	0.08	0.04	0.10	$m_{28}$	0.05	0.76	0.04	0.04	0.02	0.02	0.07
$m_{14}$	0.50	0.12	0.05	0.11	0.08	0.04	0.10	$m_{29}$	0.52	0.11	0.04	0.11	0.08	0.04	0.10
$m_{15}$	0.49	0.13	0.05	0.11	0.07	0.05	0.10	$m_{30}$	0.10	0.52	0.05	0.11	0.08	0.04	0.10

## Data Availability

Data is contained within the article.
